# A Service Evaluation of Triangle of Sleep: A Pilot Guided Self-Help Programme for Insomnia in the East Kent Neuropsychiatry Service

**DOI:** 10.1192/bjo.2026.11584

**Published:** 2026-06-30

**Authors:** Faizaan Syed, Rafey Faruqui, Alan Dunlop

**Affiliations:** Kent and Medway Mental Health NHS Trust, Ashford, United Kingdom

## Abstract

**Aims:**

Insomnia is common in people with neuropsychiatric conditions and causes distress, cognitive difficulties, and functional impairment. Although NICE recommends behavioural interventions such as CBT-I as first-line treatment, no structured non-pharmacological insomnia programme currently exists within neuropsychiatry services in Kent. To address this, we developed the Triangle of Sleep: a clinician-led, structured psychoeducationprogramme delivered in a guided self-help format, integrating sleep hygiene, daytime routine restructuring, and mindfulness body scanning.

**Methods:**

Patients were invited to three weekly 1-hour clinician-led online group sessions combining psychoeducation, discussion, and reflective between-session tasks. Outcomes were measured using the Pittsburgh Sleep Quality Index (PSQI) at baseline and 2-month follow-up. Demographics (age, gender), diagnoses, and baseline sedative/activating medications were recorded. Two cohorts have completed the programme (October 2025; January 2026), with a final cohort scheduled for March 2026.

**Results:**

Cohort 1 (n=3):

Patient 1: 41-year-old female with functional neurological disorder, non-epileptic attack disorder, chronic fatigue syndrome, fibromyalgia, migraine, and past depression/anxiety (pregabalin, nortriptyline, fluoxetine, tapentadol). Baseline PSQI showed poor sleep maintenance, prolonged wake times, and frequent nocturnal symptoms (pain, nocturia, jerky leg movements). Post-intervention, sleep duration increased 4→5 hours and wake time shifted earlier (12pm→11am). Sleep quality remained “fairly poor,” but she reported “slight improvement.” Jerky movements and nocturnal confusion reduced from “≥3 times/week” to “1–2 times/week.”

Patient 2: 54-year-old female with functional neurological disorder, fibromyalgia, chronic fatigue syndrome, migraine, depression/anxiety features, and OCD (no sedative/psychotropic medication). Baseline PSQI indicated fragmented sleep, contributed by pain and nocturia (≥3times/week). After the programme, sleep latency reduced (20→15 min), sleep duration increased (8→8.5 hours), and subjective sleep quality improved (fairly bad to fairly good), though pain/nocturia persisted.

Patient 3: 36-year-old male with relapsing-remitting multiple sclerosis and mixed anxiety/depressive disorder (gabapentin, sertraline, carbamazepine, baclofen) did not complete PSQI but reported improved sleep routine/initiation and reduced daytime cognitive fatigue.

Cohort 2 (n=8): baseline data collected; follow-up PSQI due March 2026.

**Conclusion:**

This is the first clinician-led structured psychoeducation sleep programme in Kent’s neuropsychiatry service. Early findings suggest feasibility, acceptability, and potential benefit for sleep onset/maintenance and subjective sleep quality. PSQI data highlight persistent contributors (pain, nocturia, motor symptoms) needing integrated management alongside behavioural input. In Patient 1, improvements in jerky movements and nocturnal confusion coincided with concurrent attendance to the non-epileptic attack disorder group in our service, suggesting possible synergistic benefit. Future work could adapt this p into a web-based platform with AI-support to improve scalability where specialist sleep provision is limited.

